# Antimicrobial Resistance Profiles of Gram-Negative Bacteria Isolated from Saker Falcons (*Falco cherrug*) in Western Romania

**DOI:** 10.3390/antibiotics15040400

**Published:** 2026-04-15

**Authors:** Daiana-Ionela Cocoș, Oana-Maria Boldura, Eugenia Dumitrescu, Răzvan-Tudor Pătrînjan, Florin Muselin, Diana Brezovan, Janos Degi, Romeo Teodor Cristina

**Affiliations:** 1 Doctoral School “Veterinary Medicine”, University of Life Sciences “King Mihai I” from Timişoara, Calea Aradului 119, 300645 Timişoara, Romania; razvan.patrinjan.fmv@usvt.ro; 2 Department of Pharmacy and Pharmacology, Faculty of Veterinary Medicine, University of Life Sciences “King Mihai I” from Timişoara, Calea Aradului 119, 300645 Timisoara, Romania; 3 Department of Biochemistry, Faculty of Veterinary Medicine, University of Life Sciences “King Mihai I” from Timişoara, Calea Aradului 119, 300645 Timisoara, Romania; 4 Department of Animal Production and Veterinary Public Health, Faculty of Veterinary Medicine, University of Life Sciences “King Mihai I” from Timişoara, Calea Aradului 119, 300645 Timisoara, Romania; 5 Department of Toxicology, Faculty of Veterinary Medicine, University of Life Sciences “King Mihai I” from Timişoara, Calea Aradului 119, 300645 Timisoara, Romania; 6 Department of Histology and Cell Biology, Faculty of Veterinary Medicine, University of Life Sciences “King Mihai I” from Timişoara, Calea Aradului 119, 300645 Timisoara, Romania; 7 Department of Infectious Diseases and Preventive Medicine, Faculty of Veterinary Medicine, University of Life Sciences “King Mihai I” from Timişoara, Calea Aradului 119, 300645 Timisoara, Romania

**Keywords:** antimicrobial resistance, *Enterobacteriaceae*, *Falco cherrug*, One Health, Romania

## Abstract

**Background/Objectives**: The Saker Falcon (*Falco cherrug*) is an endangered raptor species of ecological and conservation relevance. Despite its status, data regarding its microbiota and the prevalence of antimicrobial resistance (AMR) remain scarce, especially in Eastern Europe. This single-facility study aims to investigate the phenotypic and genotypic AMR profiles of Gram-negative bacteria isolated from captive Saker Falcons in Western Romania. **Methods**: Freshly voided fecal droppings were collected non-invasively from 40 clinically healthy Saker Falcons. Bacterial identification was performed using selective media and the VITEK^®^ 2 system. Antimicrobial susceptibility testing (AST) was conducted on a representative subset of 12 isolates. Selected resistance-associated genes were screened by conventional PCR. **Results**: *Escherichia coli* was the most prevalent 60% (n = 24/40), followed by *Hafnia alvei* 10% (n = 4/40) and *Pseudomonas* spp. 10% (n = 4/40). AST revealed phenotypic resistance among *Enterobacteriaceae* primarily to ampicillin 20% (n = 2/10), tetracycline 20% (n = 2/10), fluoroquinolones and sulfonamides 10% (n = 1/10), while susceptibility to imipenem 90% (n = 9/10) and gentamicin 90% (n = 9/10) remained high. The targeted resistance-associated genes were detected in selected phenotypically resistant isolates. PCR screening detected *blaZ* and *ampC* in 62.5% (n = 5/8) of tested isolates, *blaOXA-61* in 37.5% (n = 3/8), *blaOXA-51* in 25% (n = 2/8), *tetK* in 37.5% (n = 3/8), and *gyrA* in 12.5% (n = 1/8). The isolate used as the negative control, pansusceptible in AST, was confirmed negative for all targeted genes. **Conclusions**: This single-facility study provides baseline data on AMR traits in Gram-negative bacteria associated with Saker Falcons in Western Romania. Given the limited scale and isolate-based design of the study, the findings should be interpreted cautiously, but they support further investigation of wildlife-associated AMR within a One Health context.

## 1. Introduction

**Figure 1:**
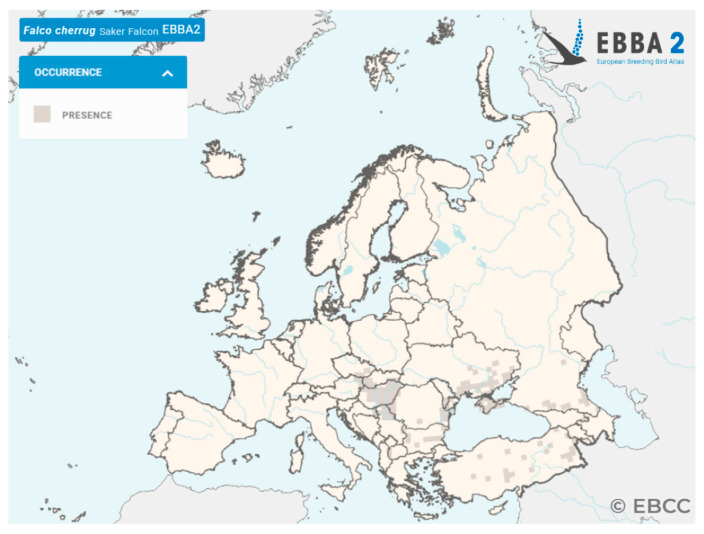
Distribution and occurrence of the Saker Falcon (*Falco cherrug*) in Europe according to the European Breeding Bird Atlas 2 (EBBA2) [1].

The Saker Falcon (*Falco cherrug* Gray, 1834), a large diurnal raptor of the family *Falconidae*, is widely distributed across the Palearctic map, from Eastern Europe and the Middle East to Central and East Asia. Across Eastern and Central Europe, breeding populations have been reported in Hungary, Romania, Bulgaria, Ukraine, and Turkey, as illustrated in Figure 1 [1].

Recently, the Saker Falcon population in Western Romania has shown a significant recovery, primarily due to conservation efforts involving the installation of artificial nest boxes on high-voltage electricity pylons [2]. According to the European Red List of Birds, the breeding population is estimated at 430–630 pairs, with the largest populations occurring in Hungary, Ukraine, and Slovakia, whereas small and declining populations persist in Romania, Bulgaria, and Serbia [3].

In Central Europe, Hungary represents an important source population for the regional expansion of this species, including toward Western Romania, and breeding success has been shown to depend strongly on habitat quality and the availability of safe nesting sites, including artificial structures [4,5,6].

Despite local recovery trends, the species remains affected by important conservation pressures in other parts of its range, including habitat loss and illegal trapping [7].

Its populations have declined during recent decades due to habitat loss, prey depletion, electrocution, and illegal trapping, leading to its classification as endangered by the International Union for Conservation of Nature (IUCN) [8,9]. The species is of ecological and cultural significance, particularly through its long association with falconry, where birds are frequently bred, traded, and maintained in captivity [10]. Studies on Saker Falcons have described wide foraging movements and have established clinical reference values useful to avian medicine [11,12]. Captive falcons used in falconry or rehabilitation may be exposed to diverse microbial communities, parasites, and environmental contaminants, and surveys from Europe and the Arabian Peninsula have reported parasitic, fungal and bacterial infections, as well as heavy metal exposure in these birds [13,14,15]. Opportunistic pathogens such as *Aspergillosis* and *Caryospora cherrughi* infection have been documented in *F. cherrug* and hybrid falcons [14,16], while microbiological screening has revealed the presence of opportunistic and potentially pathogenic bacteria, including *Escherichia coli*, *Citrobacter freundii*, *Klebsiella pneumoniae*, *Pseudomonas aeruginosa*, and *Staphylococcus aureus*, often in asymptomatic carriers [17]. Physiological and environmental stressors, including nutritional imbalance, contaminants, and human handling, may further impair immunity and facilitate bacterial colonization [18,19].

Recent advances in genetic research have provided insights into the evolutionary and molecular features of *Falco cherrug* [20,21], yet relatively little is known about its microbiota and the occurrence of antimicrobial resistance (AMR) in associated bacterial populations. Raptors are recognized as important sentinels of environmental health and potential reservoirs of antimicrobial-resistant microorganisms. Their diet, which includes small mammals and birds from agricultural landscapes, and their interaction with human activities place them at the interface of wildlife, domestic animals, and human health. Consequently, the detection of resistant Gram-negative bacteria in raptors is increasingly regarded as an early warning signal within the One Health framework [4,22,23].

Among Gram-negative bacteria, members of the *Enterobacteriaceae* family and related genera are of particular concern, as they harbor genes conferring resistance to β-lactams, tetracyclines, fluoroquinolones, and other clinically relevant antimicrobial classes. Monitoring these pathogens in wild and captive raptors can help understand the environmental dissemination of resistance determinants. Previous studies have emphasized the need for integrated surveillance linking wildlife microbiology and veterinary diagnostics to broader AMR control strategies [13,14,24,25].

The present study investigates the phenotypic and genotypic AMR profiles of Gram-negative isolates recovered from fecal samples of Saker Falcons maintained in captivity. By combining phenotypic and molecular approaches, this work aims to provide baseline data on antimicrobial resistance in *Falco cherrug* and to contribute to the broader discussion of wildlife-associated AMR within a One Health framework.

## 2. Results

### 2.1. Bacterial Isolation and Identification

A total of 40 bacterial isolates recovered from fresh fecal droppings collected non-invasively from clinically healthy Saker Falcons (*Falco cherrug*) were subjected to bacteriological examination. Gram-negative bacteria were successfully isolated and identified using the VITEK^®^ 2 automated system.

**Figure 2:**
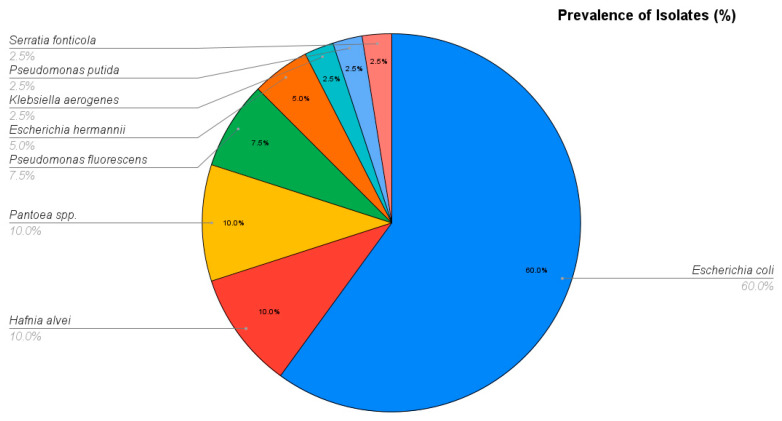
Prevalence and identification of Gram-negative bacteria isolated from Saker Falcon.

The most prevalent species was *Escherichia coli*, identified in 24 out of 40 samples, representing an isolation rate of 60.0%. Other members of the *Enterobacteriaceae* family were detected at lower frequencies, including *Hafnia alvei* 10.0% (n = 4/40), *Pantoea* spp. 10.0% (n = 4/40), and *Escherichia hermannii* 5.0% (n = 2/40). Less frequently isolated pathogens included *Serratia fonticola* and *Klebsiella aerogenes*, each detected in single samples 2.5% (n = 1/40). Additionally, *Pseudomonas* species (*P. fluorescens* and *P. putida*) were identified in 10.0% (n = 4/40) of the samples combined, as detailed in Figure 2.

The automated identification process demonstrated high reliability. The probability of identity for the vast majority of isolates ranged from 95% to 99%, corresponding to an “Excellent Confidence” level according to the VITEK^®^ 2 system specifications. Only two isolates (*S. fonticola* and *K. aerogenes*) were identified with a “Good Confidence” level (91%), which is considered sufficient for diagnostic accuracy in veterinary clinical microbiology.

The detailed identification data for each of the 40 isolates, including VITEK^®^ 2 probability levels and isolation media, are provided in Table S1.

### 2.2. Phenotypic Antimicrobial Resistance Profiles

From the total bacterial collection, a representative subset of 12 isolates was selected for detailed AST. This subset contains 10 isolates belonging to the *Enterobacteriaceae* family (predominantly *Escherichia coli*, alongside *Hafnia alvei* and *Pantoea* spp.) and 2 isolates of *Pseudomonas* spp. (*P. fluorescens*).

Among *Enterobacteriaceae*, resistance was predominantly associated with β-lactam antibiotics. Ampicillin resistance was detected in 20% (n = 2/10) of the isolates, whereas 60% (n = 6/10) were classified as susceptible. Ticarcillin/clavulanic acid showed high activity, with 80% (n = 8/10) of isolates categorized as susceptible and only 10% (n = 1/10) as resistant. The first-generation cephalosporin cefalotin showed limited efficacy, with 10% (n = 1/10) resistant, 30% (n = 3/10) intermediate, and 20% (n = 2/10) susceptible isolates. For cefalexin, 70% (n = 7/10) of isolates were classified as susceptible, while 20% (n = 2/10) exhibited phenotypic resistance. In contrast, third- and fourth-generation cephalosporins, including cefoperazone, ceftiofur, and cefquinome, demonstrated improved efficacy, with 60% (n = 6/10) of *Enterobacteriaceae* isolates characterized as susceptible.

Carbapenem susceptibility remained high among *Enterobacteriaceae*, as 90% (n = 9/10) of isolates were susceptible to imipenem. Aminoglycoside testing revealed 70% susceptibility to neomycin and 90% (n = 9/10) to gentamicin. Resistance to quinolones and fluoroquinolones (flumequine, enrofloxacin, and marbofloxacin) was low, with each agent showing 10% (n = 1/10) resistance and 50% (n = 5/10) susceptibility among *Enterobacteriaceae* isolates. Tetracycline showed moderate activity, with 40% (n = 4/10) of isolates classified as susceptible and 20% (n = 2/10) as resistant. For sulfonamides, trimethoprim/sulfamethoxazole exhibited 80% (n = 8/10) susceptibility and 10% (n = 1/10) resistance among isolates.

According to the predefined multidrug resistance (MDR) criterion (≥3 antimicrobial classes), MDR was observed in *Hafnia alvei* and *Escherichia hermannii* isolates, which exhibited simultaneous resistance to multiple antimicrobial classes, including penicillins, cephalosporins, and tetracyclines. This multidrug-resistant profile highlights the clinical relevance of the bacterial flora carried by these raptors.

The *Pseudomonas* spp. (n = 2) and *Klebsiella aerogenes* (n = 1) isolates displayed susceptibility profiles consistent with their known intrinsic resistance characteristics. For *K. aerogenes*, the isolate was classified as resistant to ampicillin and first-generation cephalosporins, a result validated by the VITEK^®^ 2 Therapeutic Resistance Monitoring (TRM) system due to the species’ constitutive chromosomal *ampC* expression. In the case of *Pseudomonas* spp., certain antibiotics yielded ‘Insufficient Evidence’ (IE) messages; this aligns with EUCAST [26] observations where clinical breakpoints are not always established for non-aeruginosa *Pseudomonas* spp., although their phenotypes remain consistent with intrinsic mechanisms, such as efflux pumps and *AmpC* β-lactamases. These findings reflect inherent biological traits rather than acquired resistance. Nevertheless, both isolates were resistant 100% (n = 2/2) to ticarcillin/clavulanic acid and showed intermediate susceptibility 100% (n = 2/2) to imipenem.

Overall, the phenotypic resistance data indicate a higher frequency of resistance to older β-lactam antibiotics among *Enterobacteriaceae*, whereas carbapenems retained high efficacy against both *Enterobacteriaceae* and *Pseudomonas* spp. Distinct resistance patterns between enteric fermenters and non-fermenting species are detailed in Table 1 and Table 2 and provide the basis for the subsequent analysis of antimicrobial resistance genes.

### 2.3. Genotypic Antimicrobial Resistance Profiles

Isolates exhibiting phenotypic resistance in AST were subjected to molecular confirmation. The number of isolates screened for each gene corresponded to the respective resistance phenotype observed in VITEK^®^ 2 testing.

The tetracycline resistance gene *tetK* was investigated for isolates phenotypically resistant to tetracycline. The isolates with tetracycline resistance yielded the expected 360 bp amplicon, indicating that the phenotypic resistance profiles are genotypically supported by the detection of the specific resistance determinants.

**Figure 3:**
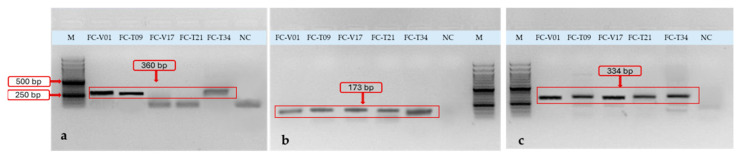
Agarose gel electrophoresis of PCR products showing amplification of selected antimicrobial resistance genes in Gram-negative isolates from Saker Falcons (*Falco cherrug*). Lane M: 50 bp DNA Ladder RTU (Simply). (**a**) Lanes FC-V01, FC-T09, FC-T34: amplification of the *tetK* gene, showing distinct bands at approximately 360 bp; (**b**) Lanes FC-V01, FC-T09, FC-V17, FC-T21, FC-T34: detection of the *blaZ* gene with an expected amplicon size of 173 bp; (**c**) Lanes FC-V01, FC-T09, FC-V17, FC-T21, FC-T34: amplification of the *ampC* gene with bands corresponding to ~334 bp. Lane NC: internal negative control (DNA from pansusceptible isolate), showing absence of amplification. Band sizes were estimated by comparison with the 50 bp molecular weight marker.

Notably, isolate FC-T34, classified as intermediately susceptible in the phenotypic AST, produced a distinct PCR amplicon corresponding to the targeted resistance gene (Figure 3a, lane 5), indicating that the resistance determinant was present despite the absence of a fully resistant phenotype.

β-lactam resistance determinants were analyzed in isolates displaying reduced susceptibility to penicillins and/or early-generation cephalosporins. Amplification of *blaZ* (173 bp) and *ampC* (334 bp) was detected in all five resistant isolates. When calculated relative to the total number of isolates subjected to AST, *blaZ* and *ampC* were present in 62.5% (n = 5/8) of tested isolates. Class D β-lactamase genes were identified at lower frequencies.

The *blaOXA-61* gene (280 bp) was detected in three isolates (37.5%, n = 3/8), while *blaOXA-51* (353 bp) was identified in two isolates (25%, n = 2/8). Importantly, despite the presence of *blaOXA* genes, none of these isolates exhibited carbapenem resistance, consistent with the high imipenem susceptibility observed phenotypically.

Fluoroquinolone-associated resistance was investigated by amplification of the quinolone resistance-determining region of *gyrA*. A specific 626 bp amplicon was detected in one isolate (12.5%, n = 1/8) exhibiting reduced susceptibility to fluoroquinolones. No amplification was observed in phenotypically susceptible isolates.

**Figure 4:**
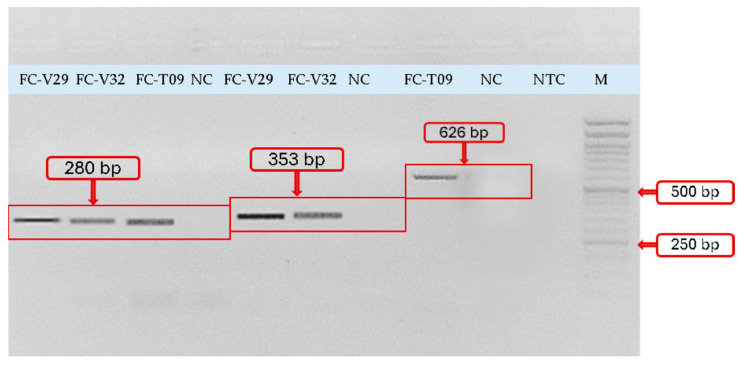
Agarose gel electrophoresis showing amplification of fluoroquinolone- and OXA-type β-lactamase-associated genes in isolates from Saker Falcons (*Falco cherrug*). Lane M: 50 bp DNA Ladder RTU (Simply). Lanes FC-V29, FC-V32, FC-T09: blaOXA-61 amplicon (~280 bp). Lanes FC-V29, FC-V32: blaOXA-51 amplicons (~353 bp). Lane FC-T09: amplification of the *FQgyrA* fragment with an expected size of 626 bp. Lane NC: internal negative control (pansusceptible isolate DNA), showing no amplification. Lane NTC: non-template control, confirming absence of reagent contamination. Fragment sizes were determined relative to the 50 bp ladder.

Overall, the distribution of resistance genes among tested isolates was as follows: *tetK* (37.5%), *blaZ* (62.5%), *ampC* (62.5%), *blaOXA-61* (37.5%), *blaOXA-51* (25.0%), and *gyrA* (12.5%) (Figure 3 and Figure 4, see also Figures S1 and S2 for original gel images in Supplementary Materials). The Quinolone Resistance-Determining Regions (QRDR) fragment of *gyrA* was successfully amplified in the phenotypically non-susceptible isolate. However, PCR amplification alone does not confirm the presence of resistance-associated mutations; sequencing is required for definitive confirmation.

The integration of phenotypic and genotypic data revealed that the observed resistance profiles were consistent with the presence of the identified resistance determinants. Table 3 summarizes these findings for the most representative isolates, highlighting the complex resistome of Gram-negative bacteria carried by captive Saker Falcons. Notably, multi-resistant isolates such as *Hafnia alvei* and *Escherichia hermannii* harbored multiple gene clusters, including *blaZ*, *ampC*, and *tetK*, which explains their resistance to penicillins, cephalosporins, and tetracyclines.

## 3. Discussion

The present study highlights the phenotypic and genotypic AMR profiles of Gram-negative bacteria isolated from the endangered Saker Falcon (*Falco cherrug*) in Western Romania. To our knowledge, this is one of the first investigations targeting this specific raptor population in the region, contributing valuable data to the limited body of literature regarding the microbiota of captive falcons in Eastern Europe. The recovery of AMR isolates from these sentinel birds underscores the growing concern of environmental pollution with resistance determinants, highlighting the relevance of raptors within the One Health framework.

### 3.1. Bacterial Prevalence and Spectrum

Our findings confirm the presence of *Enterobacteriaceae* in the gastrointestinal tract of clinically healthy captive Saker Falcons, particularly *Escherichia coli*, which exhibits multiple AMR profiles. The high prevalence of *E. coli* (60%, n = 24/40) found in our samples aligns with previous research on birds of prey, where this bacterium is frequently reported as a dominant component of the cloacal microbiota in both wild and captive individuals [27,28].

In a similar study conducted on captive falcons in Saudi Arabia and Egypt, Salah-Eldein et al. (2025) [29] reported an *E. coli* prevalence of 11.77% (n = 4/34) in fecal samples and 21.43% (n = 6/28) in oropharyngeal swabs. Similarly, Vidal et al. (2017) [30] identified *E. coli* as the most frequent isolate at 35% (n = 160/457) in diseased free-living raptors in Spain. The detection of these bacteria in our study, even in the absence of clinical symptoms, suggests that Saker Falcons may act as asymptomatic reservoirs.

The detection of MDR profiles of *Hafnia alvei* and *Escherichia hermannii* is noteworthy, as these isolates exhibited resistance to at least three antimicrobial categories. This finding suggests that captive Saker Falcons may harbor Gram-negative bacteria with increased resilience to multiple antimicrobial classes in Western Romania. Similar MDR profiles have been documented in other wildlife species from the same region, such as fallow deer [31], supporting the broader relevance of wildlife-associated antimicrobial resistance in this area. However, the present study was not designed to investigate transmission routes or the dissemination of resistance genes between domestic and wild animal populations, and such interpretations should therefore be made with caution.

The detection of multiple Gram-negative taxa in our study, such as *Hafnia alvei* (10%, n = 4/40) and *Pantoea* spp. (10%, n = 4/40), further indicates that the cultivable bacterial population recovered from the fecal material was taxonomically heterogeneous. This observation is consistent with the expected diversity of enteric bacteria associated with carnivorous birds, whose microbiota may include opportunistic or potentially pathogenic taxa without necessarily being associated with clinical disease [32].

### 3.2. Phenotypic Antimicrobial Resistance

The phenotypic resistance patterns observed in this study provide baseline data on resistant Gram-negative isolates recovered from captive Saker Falcons in Romania. Our findings of a 20% (n = 2/10) resistance rate to penicillins and the same to tetracycline among *Enterobacteriaceae* are in line with broader European reports showing that these antibiotic classes are among those most frequently associated with resistance in wild bird isolates [33].

A notable finding was the 10% (n = 1/10) resistance to quinolones. As fluoroquinolones are considered “critically important antimicrobials” and are frequently used in avian medicine, the presence of this phenotype is relevant from both veterinary and public health perspectives [29,34]. However, because no data on previous antimicrobial treatments or specific exposure sources were available, the origin of this resistance could not be determined in the present study. Compared to diseased raptors in Spain, where resistance to amoxicillin was observed at higher levels [30], the isolates examined in our study showed lower resistance frequencies, although AMR traits remained detectable within the tested subset. The high sensitivity to imipenem (90%, n = 9/10) suggests that carbapenem-resistant *Enterobacteriaceae* (CRE) were not prominent among the isolates tested in this specific falcon population, unlike in other European wild bird species [28].

### 3.3. Genotypic Characterization

The genotypic analysis in this study focused on a specific set of markers to elucidate the mechanisms underlying the observed resistance in Saker Falcon isolates. We investigated the presence of *tetK* (tetracycline resistance), *blaZ* (β-lactamase associated with penicillin resistance), *ampC* (cephalosporinase), *blaOXA-61*, *blaOXA-51* (class D β-lactamases), and the *gyrA* region associated with fluoroquinolone resistance.

Although *tetK* and *blaZ* are more commonly reported in Gram-positive bacteria, these genes have also been identified in environmental and wildlife-associated Gram-negative isolates, likely through horizontal gene transfer. Their inclusion in the present study aimed to explore potential cross-species dissemination of resistance determinants in a mixed ecological environment, such as captive wildlife facilities [35].

The investigation of the *blaZ* and *ampC* genes is particularly relevant given the 20% phenotypic resistance to ampicillin observed in our *Enterobacteriaceae* isolates. While *blaZ* is classically associated with staphylococcal penicillin resistance, its screening in the context of raptor microbiota is crucial, as these birds often harbor a mix of Gram-positive and Gram-negative bacteria, facilitating potential horizontal gene transfer [29,34]. The focus on *ampC* and Class D β-lactamases (*blaOXA-61* and *blaOXA-51*) reflects a proactive approach to extended-spectrum resistance monitoring. Although these OXA-type carbapenems are more frequently reported in *Acinetobacter* or *Campylobacter* species, their surveillance in apex predators like the Saker Falcon is relevant for monitoring the occurrence of such determinants in wildlife-associated bacterial isolates [28,33].

Regarding tetracycline resistance, we targeted the *tetK* gene, an efflux pump mechanism. In European wild bird populations, tetracycline resistance is often mediated by various *tet* genes, and the detection of such markers in the Western Romanian population aligns with the high prevalence of tetracycline resistance reported in broader European systematic reviews [33].

The detection of the resistance gene in isolate FC-T34, which exhibited only intermediate susceptibility in the phenotypic AST, highlights a known genotype–phenotype discordance frequently reported in antimicrobial resistance studies. The presence of resistance determinants does not necessarily translate into a fully resistant phenotype, as gene expression may be modulated by regulatory elements, transcriptional activity, or environmental conditions influencing gene activation. Moreover, certain resistance genes may remain weakly expressed or require additional chromosomal mutations to achieve clinically relevant levels of resistance. Similar observations have been discussed in detail by Martínez et al. (2015) [36] in their analysis of environmental resistomes, by Munita and Arias (2016) [37] in their review of AMR mechanisms, and by Van Belkum and Dunne (2013) [38] in the context of genotype–phenotype discrepancies in antimicrobial susceptibility testing.

A key finding of our study is the detection of the *gyrA* region in the *E. hermannii* isolate, which is consistent with its phenotypic resistance to flumequine, enrofloxacin, and marbofloxacin. This confirms that fluoroquinolone resistance in these raptors is likely mediated by chromosomal mutations in the QRDR [30]. Further sequencing of the QRDRs is necessary to confirm the presence of specific mutations associated with fluoroquinolone resistance.

### 3.4. Ecological and Conservation Implications

The Saker Falcon is an endangered species with a fragile population status in Romania and the broader Pan-Pannonian region. The health status of these birds is crucial for conservation efforts. Fântână et al. (2025) [39] emphasized the importance of monitoring parameters for the remaining breeding pairs in Southern Romania. Infectious diseases and the carriage of MDR bacteria represent cryptic threats that could compromise rehabilitation success or breeding programs. As noted by Giacopello et al. (2016) [28], wildlife rescue centers and captive breeding facilities must implement strict hygiene protocols to prevent the amplification of resistant strains. Although the present study did not include parallel sampling from humans, domestic animals, or environmental matrices, the detection of resistant Gram-negative bacteria in captive raptors remains relevant to the One Health perspective, as these birds occupy an interface between wildlife, husbandry-associated environments, and human handling. In this context, our findings should be interpreted as baseline wildlife-associated AMR data rather than as direct evidence of transmission across sectors.

### 3.5. Limitations and Future Directions

This study has limitations, primarily the restriction to a single facility, which may not fully reflect the AMR status of wild Saker Falcon populations in Romania. However, given the species’ rarity, these data provide a valuable regional baseline. Future research should aim to compare captive and wild populations and should incorporate broader molecular approaches, including metagenomic analysis, the investigation of mobile genetic elements, and amplicon sequencing, in order to better understand the dissemination of AMR at the human–wildlife interface. Furthermore, our findings highlight the value of routine microbiological surveillance in falconry facilities, serving not only as a diagnostic tool but also as a proactive management strategy. Integrating health screening with genomic profiling in captive breeding programs may help improve the evaluation of individuals intended for reinforcement or falconry, while also supporting monitoring for potential pathogen transmission [32,40,41]. Such a multidisciplinary approach may help maintain captive populations in good health and genetic robustness, and reduce the risk of introducing resistant pathogens into the wild during reintroduction programs or the movement of birds.

## 4. Materials and Methods

### 4.1. Sampling

Freshly voided fecal droppings were collected non-invasively from 40 Saker Falcons housed in a single falconry facility, positioned near Timișoara, Romania. Sampling was performed using Copan^®^ sterile transport tubes with eSwab LQ Amies (urethral flocked applicator, customized for MLS; eSwab 1 mL minitip progressive FLOQSwab BP80 MLS) (Copan Diagnostics, Brescia, Italy). Samples were transported under refrigerated conditions (4 °C) and processed within 24 h of collection. All falcons included in this study were clinically healthy at the time of sampling. The study was therefore designed as an isolate-based analysis, and the bacterial isolate was considered the analytical unit.

### 4.2. Bacterial Isolation and Culture Conditions

For bacterial isolation, the collected fecal material was inoculated onto both Violet Red Bile Glucose (VRBG) agar (Oxoid, Basingstoke, UK) and Tryptone Bile X-Glucuronide (TBX) agar (Oxoid, Basingstoke, UK). These media are recommended for selective isolation of *Enterobacteriaceae* and *Escherichia coli*, respectively, according to ISO standards (no. 16649-2:2001 and no. 21528-2:2017) [42,43]. Plates were incubated aerobically at 37 °C for 24 h. After incubation, multiple representative colonies showing distinct morphologies were selected to recover phenotypically distinct Gram-negative isolates for further characterization. A total of 40 bacterial isolates were successfully recovered and stabilized: 16 isolates from TBX plates and 24 isolates from VRBG plates. The isolates were codified based on the selective media used for recovery: those labelled with the prefix ‘V’ (e.g., FC-V01) originated from VRBG agar, while those labelled with ‘T’ (e.g., FC-T05) were recovered from TBX agar.

### 4.3. Bacterial Identification

Isolates were identified using the VITEK^®^ 2 automated system (bioMérieux, Marcy-l’Étoile, France), performed with VITEK^®^ 2 GN ID cards (REF 21341), designed for the identification of *Enterobacteriaceae* and selected non-fermenting Gram-negative organisms. Individual identification reports were generated for each of the 40 isolates. The VITEK^®^ 2 system is widely used in both clinical and veterinary microbiology, providing rapid and standardized results.

### 4.4. Antimicrobial Susceptibility Testing

AST was performed using the VITEK^®^ 2 AST system (bioMérieux, Marcy-l’Étoile, France). From the total collection of Gram-negative isolates, a representative subset of 12 isolates was selected for AST in order to capture phenotypic and taxonomic diversity observed on primary culture plates while avoiding redundant testing of colonies with similar morphology and identification profiles. This subset-based approach was intended to provide baseline information on resistance profiles among distinct cultivable isolates and not to estimate isolate-level prevalence across the entire collection. Each isolate was tested using VITEK^®^ 2 AST-GN96 cards (REF 421849), validated for Gram-negative bacteria of veterinary relevance, according to the manufacturer’s instructions. MDR was defined as resistance to at least three antimicrobial classes. Interpretation of susceptibility profiles was performed in accordance with the Clinical and Laboratory Standards Institute (CLSI) and the European Committee on Antimicrobial Susceptibility Testing (EUCAST) [26,44].

### 4.5. Molecular Confirmation by PCR

Molecular confirmation of phenotypically detected antimicrobial resistance was performed by conventional PCR targeting selected resistance determinants in isolates exhibiting reduced susceptibility in the VITEK^®^ 2 antimicrobial susceptibility testing. Genomic DNA was extracted from overnight bacterial cultures by thermal lysis. Briefly, a single colony was suspended in 1 mL sterile nuclease-free water and centrifuged at 12,000× *g* for 5 min. The pellet was resuspended in 200 µL nuclease-free water, incubated at 95 °C for 10 min to ensure complete cell lysis, and centrifuged again at 12,000× *g* for 5 min. The supernatant containing genomic DNA was collected and stored at −20 °C until further use.

DNA concentration and purity were assessed using a NanoDrop™ 8000 spectrophotometer (Thermo Fisher Scientific, Waltham, MA, USA). Only DNA samples with A260/A280 ratios between 1.8 and 2.0 and concentrations ≥ 25 ng/µL were used for amplification. PCR assays were performed to confirm the presence of resistance genes corresponding to the observed phenotypic profiles.

The following genes were investigated: *tetK* (tetracycline resistance), *blaZ* (β-lactamase associated with penicillin resistance), *ampC* (cephalosporinase), *blaOXA-61* and *blaOXA-51* (class D β-lactamases), and *gyrA* (fluoroquinolone resistance-associated region).

Amplification reactions were carried out in a final volume of 25 µL containing 12.5 µL GoTaq^®^ Green Master Mix (Promega, Madison, WI, USA), 1 µL of each primer (20 pmol), 1 µL template DNA (~25–50 ng), and nuclease-free water to volume. PCR amplification was performed using an Eppendorf Mastercycler^®^ Pro S (Eppendorf, Hamburg, Germany) under the following cycling conditions: initial denaturation at 95 °C for 5 min; 35 cycles of denaturation at 95 °C for 30 s, annealing at gene-specific temperatures (Table 4) for 30 s, and extension at 72 °C for 45 s; followed by a final extension at 72 °C for 5 min.

Each PCR run included an internal negative control consisting of DNA extracted from an isolate phenotypically susceptible to all tested antibiotics. PCR screening was restricted to isolates showing phenotypic resistance or reduced susceptibility to the corresponding antimicrobial classes and was therefore intended as targeted molecular confirmation rather than comprehensive resistome profiling.

PCR products were resolved by electrophoresis on 1.5% agarose gels prepared in 1× TAE buffer and stained with ethidium bromide. Electrophoresis was conducted at 90–100 V for approximately 60 min. A 50 bp DNA Ladder RTU (Simply, Cat. No. SD012-R500) was used as a molecular size marker, enabling precise sizing of amplicons ranging from 150 to 700 bp. Amplified fragments were visualized under UV illumination using a Gel Doc™ XR imaging system (Bio-Rad Laboratories, Hercules, CA, USA). Data acquisition and precise band size determination, relative to the molecular weight marker, were performed using Quantity One^®^ 1-D Analysis Software (version 4.6.5). Primer sequences, annealing temperatures, and expected amplicon sizes are presented in Table 4. The negative control for resistance genes consisted of DNA extracted from a bacterial isolate identified by the VITEK^®^ 2 automated system as pan-susceptible to all antimicrobial agents.

## 5. Conclusions

In conclusion, Saker Falcons in Romania harbored Gram-negative bacteria with phenotypic and selected genotypic traits of antimicrobial resistance, including resistance to antimicrobial classes relevant to both human and veterinary medicine. Although derived from a single-facility baseline investigation, these findings could be a primary source from Romania that supports further investigation of raptors extending the studies of wildlife-associated AMR.

## Figures and Tables

**Table 1:** Antimicrobial susceptibility testing results for bacterial isolates.

	AMP	AMC	TIC/CLA	CEX	CFL	CFP	CTF	CFQ	IPM	GEN	NEO	FLU	ENR	MAR	TET	SXT
*Hafnia alvei* FC-V01	R	R	S	R	R	S	S	S	S	S	S	S	S	S	R	S
*E. hermannii* FC-T09	R	R	I	R	S	S	S	S	S	S	S	R	R	R	R	R
*K. aerogenes* FC-V15	IR	IR	IR	IR	IR	IR	IR	IR	IR	IR	S	IR	IR	IR	IR	IR
*E. coli* FC-V17	S	S	S	S	I	S	S	S	S	S	S	S	S	S	S	S
*E. coli* FC-T21	S	S	S	S	I	S	S	S	S	S	S	S	S	S	S	S
*P. agglomerans* *FC-V25*	-	S	S	S	S	S	S	S	S	S	S	S	S	S	S	S
*Ps. fluorescens* FC-V29	IE	IE	R	IE	-	-	-	-	I	IE	-	-	-	-	IE	IE
*E. coli* FC-T33	S	-	S	S	-	-	-	-	S	S	-	-	-	-	IE	S
*E. coli* FC-T34	S	S	S	S	I	S	S	S	S	S	S	S	S	S	S	S
*E. coli* FC-V37	S	S	S	S	-	-	-	-	S	S	-	-	-	-	IE	S
*E. coli* FC-V38	S	-	S	S	-	-	-	-	S	S	-	-	-	-	IE	S
*Ps. fluorescens* FC-V32	IE	IE	R	IE	-	-	-	-	I	IE	-	-	-	-	IE	IE

Note: S = Susceptible (green); I = Intermediate (yellow); R = Resistant (red); IR= Intrinsic Resistance (gray); IE = Insufficient Evidence that species is good target for therapy (blue); AMP—ampicillin; AMC—amoxicillin–clavulanate; TIC/CLA—ticarcillin–clavulanate; CEX—cefalexin; CFL—cefalotin; CFP—cefoperazone; CTF—ceftiofur; CFQ—cefquinome; IPM—imipenem; GEN—gentamicin; NEO—neomycin; FLU—flumequine; ENR—enrofloxacin; MAR—marbofloxacin; TET—tetracycline; SXT—trimethoprim–sulfamethoxazole; “-” indicates antibiotic not tested or no interpretative result available.

**Table 2:** Antimicrobial resistance patterns across antibiotic classes in *Enterobacteriaceae* vs. *Pseudomonas* spp. isolates.

Antimicrobial Class	Antibiotic Agent	*Enterobacteriaceae* (n = 10)			*Pseudomonas* spp. (n = 2)		
		R (%)	I (%)	S (%)	R (%)	I (%)	S (%)
Penicillins	Ampicillin	20		60			
	Amoxicillin/Clavulanic Acid	20		50			
	Ticarcillin/Clavulanic Acid		10	80	100		
Cephalosporins	Cefalexin	20		70			
	Cefalotin	10	30	20			
	Cefoperazone			60			
	Ceftiofur			60			
	Cefquinome			60			
Carbapenems	Imipenem			90		100	
Aminoglycosides	Gentamicin			90			
	Neomycin			70			
Chinolone	Flumequine	10		50			
Fluoroquinolones	Enrofloxacin	10		50			
	Marbofloxacin	10		50			
Tetracyclines	Tetracycline	20		40			
Sulfonamides	Trimethoprim/Sulfamethoxazole	10		80			
Others	Florfenicol (Amphenicol Class)						
	Polymyxin B (Polypeptide Class)						

Note: S = Susceptible; I = Intermediate; R = Resistant; Percentages were calculated based on the number of isolates for which AST interpretation was available. “IE” (insufficient evidence) and “IR” (intrinsic resistance) results were excluded from percentage calculations.

**Table 3:** Phenotypic and genotypic antimicrobial resistance profiles of bacterial isolates from *Falco cherrug*.

Bacterial Isolate	Sample ID	Phenotype			Gene Detected
		Resistance (R)	Susceptibility (S)	Intermediate (I)	
*Hafnia alvei*	*FC-V01*	AMP, AMC, CEX, CFL, TET	TIC/CLA, CFP, CTF, CFQ, IPM, GEN, NEO, FLU, ENR, MAR, SXT	-	*tetK* *blaZ* *ampC*
*Ps. fluorescens*	*FC-V29*	TIC/CLA	-	IPM	*blaOXA-61 blaOXA-51*
*Ps. fluorescens*	*FC-V32*	TIC/CLA	-	IPM	*blaOXA-61 blaOXA-51*
*E. hermannii*	*FC-T09*	AMP, AMC, CEX, FLU, ENR, MAR, TET, SXT	CFL, CFP, CTF, CFQ, IPM, GEN, NEO	TIC/CLA	*tetK* *blaZ* *ampC* *blaOXA-61 gyrA*
*E. coli*	*FC-V17*	-	AMP, AMC, TIC/CLA, CEX, CFP, CTF, CFQ, IPM, GEN, NEO, FLU, ENR, MAR, TET, SXT	CFL	*blaZ,* *ampC*
*E. coli*	*FC-T21*	-	AMP, AMC, TIC/CLA, CEX, CFP, CTF, CFQ, IPM, GEN, NEO, FLU, ENR, MAR, TET, SXT	CFL	*blaZ* *ampC*
*E. coli*	*FC-T34*	-	AMP, AMC, TIC/CLA, CEX, CFP, CTF, CFQ, IPM, GEN, NEO, FLU, ENR, MAR, TET, SXT	CFL	*tetK* *blaZ* *ampC*
*E. coli*	*FC-T33*	-	AMP, TIC/CLA, CEX, IPM, GEN, SXT	-	-

Note: AMP: Ampicillin; AMC: Amoxicillin/Clavulanic acid; TIC/CLA: Ticarcillin/Clavulanic acid; CEX: Cephalexin; CFL: Cephalothin; CFP: Cefoperazone; CTF: Ceftiofur; CFQ: Cefquinome; IPM: Imipenem; GEN: Gentamicin; NEO: Neomycin; FLU: Flumequine; ENR: Enrofloxacin; MAR: Marbofloxacin; TET: Tetracycline; SXT: Sulfamethoxazole/Trimethoprim.

**Table 4:** Primer sequences used for PCR amplification of AMR genes in Gram-negative isolates from *Falco cherrug*.

Target Gene	Primer	Primer Sequence (5′–3′)	Amplicon Size (bp)	Annealing Temperature	Reference
***tetK***	*tetK* F	GTAGCGACAATAGGTAATAGT	360	49 °C	[45]
	*tetK* R	GTAGTGACAATAAACCTCCTA			
***blaZ***	*blaZ* F	ACTTCAACACCTGCTGCTTTC	173	50 °C	[45]
	*blaZ* R	TGACCACTTTTATCAGCAACC			
***ampC***	*ampC* F	ATGATGAAAAAATCGTTATGC	334	50 °C	[46]
	*ampC* R	TTGCAGCTTTTCAAGAATGC			
***blaOXA-61***	*blaOXA-61* F	GAGTATAATACAAGCGGCAC	280	56 °C	[47]
	*blaOXA-61* R	CCAATTCTTCTTGCCACTTC			
***blaOXA-51***	*blaOXA-51* F	TAATGCTTTGATCGGCCTTG	353	60 °C	[48]
	*blaOXA-51* R	TGGATTGCACTTCATCTTGG			
***FQgyrA***	*FQgyrA* F	ACGTACTGTCGGTAACAGTG	626	55 °C	[49]
	*FQgyrA* R	TTAATGATTGCCGCCGTCGG			

## Data Availability

The data supporting the findings of this study are contained within the article.

## Supplementary Materials

The following supporting information can be downloaded at: https://www.mdpi.com/article/10.3390/antibiotics15040400/s1, Table S1: Identification and origin of the 40 bacterial isolates analyzed by the VITEK^®^ 2 automated system; Figure S1: Uncropped Figure 3; Figure S2: Uncropped Figure 4.

## Abbreviations

The following abbreviations are used in this manuscript:

AMC	Amoxicillin–clavulanic acid
AMP	Ampicillin
AMR	Antimicrobial Resistance
AST	Antimicrobial Susceptibility Testing
bp	Base pairs
CEX	Cefalexin
CFL	Cefalotin
CFP	Cefoperazone
CFQ	Cefquinome
CLSI	Clinical and Laboratory Standards Institute
CRE	Carbapenem-resistant *Enterobacteriaceae*
CTF	Ceftiofur
DNA	Deoxyribonucleic Acid
ENR	Enrofloxacin
EU	European Union
EUCAST	European Committee on Antimicrobial Susceptibility Testing
FLU	Flumequine
GEN	Gentamicin
I	Intermediate (susceptibility)
IE	Insufficient Evidence (that species is a good target for therapy)
IPM	Imipenem
MAR	Marbofloxacin
MDR	Multidrug-Resistant
NEO	Neomycin
PCR	Polymerase Chain Reaction
R	Resistant
S	Susceptible
SXT	Trimethoprim–sulfamethoxazole
TAE	Tris-Acetate-EDTA (buffer)
TBX	Tryptone Bile X-glucuronide (agar)
TET	Tetracycline
TIC/CLA	Ticarcillin–clavulanic acid
TRM	Therapeutic Resistance Monitoring
UV	Ultraviolet
V	Volts
VRBG	Violet Red Bile Glucose (agar)

## References

[1] European Bird Census Council (EBCC) European Breeding Bird Atlas 2: Saker Falcon (*Falco cherrug*) Abundance Map. https://ebba2.info/maps/species/Falco-cherrug/ebba2/occurrence/.

[2] Prommer M., Hegyeli Z., Nagy A. (2025). Population Recovery and Spatial Determinants of Occupancy and Breeding Success in the Saker Falcon (*Falco cherrug*): A Study from Western Romania. Ornis Hung..

[3] (2021). BirdLife International *Falco cherrug* (Europe Assessment).

[4] Bagyura J., Haraszthy L., Szitta T., Solti B., Jánossy D., Prommer M., Fidlóczky J., Horváth M. (2025). Population Trend, Breeding Performance and Diet of Saker Falcons (*Falco cherrug*) in Hungary between 1980 and 2024. Ornis Hung..

[5] Zink R., Hohenegger J.A., Berg H.-M., Kmetova-Biro E. (2025). Population Trend and Conservation of Saker Falcon (*Falco cherrug*) in Austria (2012–2021). Ornis Hung..

[6] Chavko J., Lipták J., Gális M., Slobodník R., Prommer M. (2025). Distribution, Abundance and Reproductive Success of the Saker Falcon in Slovakia in 1976–2022. Ornis Hung..

[7] Farajli Z., Kittelberger K.D., Tanner C.J., Aghababyan K., Paposhvili N., Hakiminejad M., Karyakin I., Belik V., Şekercioğlu Ç.H., Çoban E. (2025). Status and Breeding Population of the Saker Falcon (*Falco cherrug*) in the Caucasus Ecoregion: Regional Perspectives and Conservation Challenges. Caucasiana.

[8] Sun J., Dixon A., Gu Z., Lin Z., Zhan X. (2021). Status of the Saker Falcon in China. Sci. China Life Sci..

[9] Shobrak M.Y. (2015). Trapping of Saker Falcon *Falco cherrug* and Peregrine Falcon *Falco peregrinus* in Saudi Arabia: Implications for Biodiversity Conservation. Saudi J. Biol. Sci..

[10] Petrov R., Hoareau T., Lesobre L., Andonova Y., Yarkov D., Chakarov N. (2023). Genetic Diversity and Relatedness amongst Captive Saker Falcons (*Falco cherrug*) in the Green Balkans’ Wildlife Rehabilitation and Breeding Centre in Bulgaria. Biodivers. Data J..

[11] Bold B., Rahman L., Purev-Ochir G., Saruul A., Zhan X., Dixon A. (2024). Influence of Prey Availability on the Movement Pattern of Breeding Saker Falcons (*Falco cherrug*) in Mongolia. Curr. Zool..

[12] Samour J.H., D’aloia M.A. (1996). Normal Blood Chemistry of the Saker Falcon (*Falco cherrug*). Avian Pathol..

[13] Prątnicka A., Sokół R., Iller M. (2024). Parasitic Survey of Birds of Prey Used for Falconry in Poland. Pol. J. Vet. Sci..

[14] Van Waeyenberghe L., Fischer D., Coenye T., Ducatelle R., Haesebrouck F., Pasmans F., Lierz M., Martel A. (2012). Susceptibility of Adult Pigeons and Hybrid Falcons to Experimental Aspergillosis. Avian Pathol..

[15] Zolfaghari G., Esmaili-Sari A., Ghasempouri S.M., Kiabi B.H. (2007). Examination of Mercury Concentration in the Feathers of 18 Species of Birds in Southwest Iran. Environ. Res..

[16] Alfaleh F.A., Alyousif M.S., Al-Shawa Y.R., Al-Quraishy S. (2013). *Caryospora cherrughi* sp. n. (Apicomplexa: Eimeriidae) Infecting *Falco cherrug* in Saudi Arabia. Parasitol. Res..

[17] Konstantinov A.V., Pimenov N.V., Pavlova A.V., Ivannikova R.F. (2021). Features of the Microbiota of Falconiformes Birds. IOP Conf. Ser. Earth Environ. Sci..

[18] Castilla A.M., Herrel A., Van Dongen S., Furio N., Negro J.J. (2009). Determinants of Eggshell Strength in Endangered Raptors. J. Exp. Zool. Part A Ecol. Genet. Physiol..

[19] Petrov R., Cholakova D. (2025). Oldest Known Captive Saker Falcon (*Falco cherrug cherrug*) at 31 Years Old. Biodivers. Data J..

[20] Lu J., Lu J., Li X.-F., Jiang H. (2016). Complete Mitochondrial Genome of the Saker Falcon, *Falco cherrug* (Falco, Falconidae). Mitochondrial DNA A DNA Mapp. Seq. Anal..

[21] Zhan X., Pan S., Wang J., Dixon A., He J., Muller M.G., Ni P., Hu L., Liu Y., Hou H. (2013). Peregrine and Saker Falcon Genome Sequences Provide Insights into Evolution of a Predatory Lifestyle. Nat. Genet..

[22] Cocoș D.I., Folescu M., Orășan-Alic S., Doma A.O., Dumitrescu E., Cristina R.T. (2024). Patterns of Antimicrobial Resistance in *E. coli* Isolates from Europe’s Wild Birds. Lucr. Stiintifice Med. Vet. Timis. (Sci. Pap. Vet. Med.).

[23] Cocoș D.I., Folescu M., Ardelean L.-M., Stoichescu C., Dumitrescu E., Cristina R.T. (2025). Tendințe în cercetarea RAM: O perspectivă bibliometrică asupra păsărilor domestice vs. sălbatice (2015–2025). Vet. Drug/Med. Vet..

[24] Cristina R.T. (2024). The use of antibiotics and the evolution of antibiotic resistance in animalpopulations—A review. Med. Vet./Vet. Drug.

[25] Doma A.O., Popescu R., Mituletu M., Muntean D., Degi J., Boldea M.V., Radulov I., Dumitrescu E., Muselin F., Puvaca N. (2020). Comparative Evaluation of *qnrA*, *qnrB*, and *qnrS* Genes in *Enterobacteriaceae* Ciprofloxacin-Resistant Cases, in Swine Units and a Hospital from Western Romania. Antibiotics.

[26] The European Committee on Antimicrobial Susceptibility Testing (EUCAST) Breakpoint Tables for Interpretation of MICs and Zone Diameters. https://www.eucast.org/fileadmin/eucast/pdf/breakpoints/v_15.0_Breakpoint_Tables.pdf.

[27] Khafagy A., Kamel A., Moursy M., Aidaroos N., Ahmed D. (2018). Phenotypic and Genotypic Characterization of Gram Negative Bacteria Isolated from Birds of Prey (Raptors). Suez Canal Vet. Med. J. SCVMJ.

[28] Giacopello C., Foti M., Mascetti A. (2016). Antimicrobial Resistance Patterns of *Enterobacteriaceae* in European Wild Bird Species Admitted in a Wildlife Rescue Centre. Vet. Ital..

[29] Salah-Eldein A.M., Elnoubi M.A., Medani G.G., Eidaroos N.H., Elassy N.M., Saad E.M. (2025). Isolation and Antibiotic Resistance of *Escherichia coli* and *Staphylococcus aureus* from Captive Falcons. Egypt. Acad. J. Biol. Sci. B Zool..

[30] Vidal A., Baldomà L., Molina-López R.A., Martin M., Darwich L. (2017). Microbiological Diagnosis and Antimicrobial Sensitivity Profiles in Diseased Free-Living Raptors. Avian Pathol..

[31] Tîrziu E., Bulucea A.V., Imre K., Nichita I., Muselin F., Dumitrescu E., Tîrziu A., Mederle N.G., Moza A., Bucur I.M. (2023). The Behavior of Some Bacterial Strains Isolated from Fallow Deer Compared to Antimicrobial Substances in Western Romania. Antibiotics.

[32] Ahmad A.R., Ridgeway S., Shibl A.A., Idaghdour Y., Jha A.R. (2024). Falcon Gut Microbiota Is Shaped by Diet and Enriched in *Salmonella*. PLoS ONE.

[33] Cocoș D.-I., Dumitrescu E., Muselin F., Brezovan D., Degi J., Boldura O.-M., Cristina R.T. (2025). Prevalence and Antimicrobial Resistance of *Enterobacteriaceae* in Wild Birds Across Europe: A Systematic Review. Antibiotics.

[34] Magalhães R., Tavares L., Oliveira M. (2024). Antimicrobial Resistance and Virulence Potential of Bacterial Species from Captive Birds of Prey—Consequences of Falconry for Public Health. Animals.

[35] Bryan A., Shapir N., Sadowsky M.J. (2004). Frequency and Distribution of Tetracycline Resistance Genes in Genetically Diverse, Nonselected, and Nonclinical *Escherichia coli* Strains Isolated from Diverse Human and Animal Sources. Appl. Environ. Microbiol..

[36] Martínez J.L., Coque T.M., Baquero F. (2015). What Is a Resistance Gene? Ranking Risk in Resistomes. Nat. Rev. Microbiol..

[37] Munita J.M., Arias C.A. (2016). Mechanisms of Antibiotic Resistance. Microbiol. Spectr..

[38] Van Belkum A., Dunne W.M. (2013). Next-Generation Antimicrobial Susceptibility Testing. J. Clin. Microbiol..

[39] Fântână C., Veres-Szászka J., Szabó J., Bugariu S., Todorov E., Drăgan D., Damoc D., Veres-Szászka N., Domșa C., Pui A. (2025). The Saker Falcon (*Falco cherrug*) in Southern Romania: Population, Trend and Habitat Requirements in the Breeding Season. Ornis Hung..

[40] Hoareau T.B., Barbosa A., Velkeneers X., Leveque G., Lesobre L. (2025). A Framework for Integrating Genomic Profiles into Captive Breeding and Reinforcement Programmes: A Case Study on Captive Saker Falcons. bioRxiv.

[41] Asma S.T., Imre K., Morar A., Imre M., Acaroz U., Shah S.R.A., Hussain S.Z., Arslan-Acaroz D., Istanbullugil F.R., Madani K. (2022). Natural Strategies as Potential Weapons against Bacterial Biofilms. Life.

[42] (2001). Microbiology of Food and Animal Feeding Stuffs—Horizontal Method for the Enumeration of Beta-Glucuronidase-Positive *Escherichia coli*—Part 2: Colony-Count Technique at 44 Degrees C Using 5-Bromo-4-Chloro-3-Indolyl Beta-D-Glucuronide. https://cdn.standards.iteh.ai/samples/29824/cbb8efcaa00647c3807681d77c0fb144/ISO-16649-2-2001.pdf.

[43] (2017). ISO Microbiology of the Food Chain—Horizontal Method for the Detection and Enumeration of *Enterobacteriaceae*—Part 2: Colony-Count Technique. https://cdn.standards.iteh.ai/samples/63504/5abbe17268eb4268a87a826fdd429a11/ISO-21528-2-2017.pdf.

[44] Clinical and Laboratory Standards Institute (CLSI) (2018). Performance Standards for Antimicrobial Susceptibility Testing.

[45] Quraishi A., Kaur P., Singh Sharma N., Arora A.K. (2021). Antibiotic Sensitivity Patterns in *Staphylococcus* spp. Isolated from Goat Milk in Association with Molecular Detection of Antibiotic Resistance Genes. Iran. J. Vet. Res..

[46] Pérez-Pérez F.J., Hanson N.D. (2002). Detection of Plasmid-Mediated AmpC β-Lactamase Genes in Clinical Isolates by Using Multiplex PCR. J. Clin. Microbiol..

[47] Griggs D.J., Peake L., Johnson M.M., Ghori S., Mott A., Piddock L.J.V. (2009). β-Lactamase-Mediated β-Lactam Resistance in *Campylobacter* Species: Prevalence of Cj0299 (*Bla*_OXA-61_) and Evidence for a Novel β-Lactamase in *C. jejuni*. Antimicrob. Agents Chemother..

[48] Hussein N.H., Al-Mathkhury H.J.F., Sabbah M.A. (2014). Identification of Imipenem-Resistant Genes in *Acinetobacter baumannii* Isolated from Baghdad Hospitals. J. Med. Microb. Diagn..

[49] Weigel L.M., Steward C.D., Tenover F.C. (1998). *gyrA* Mutations Associated with Fluoroquinolone Resistance in Eight Species of *Enterobacteriaceae*. Antimicrob. Agents Chemother..

