# Supplementary material for: Antimicrobial Resistance Profiles of Gram-Negative Bacteria Isolated from Saker Falcons (Falco cherrug) in Western Romania
Source: Antibiotics (Basel). 2026 Apr 15;15(4):400. doi: 10.3390/antibiotics15040400 (PMC13114167; doi:10.3390/antibiotics15040400)
Supplement: Supplementary file 1 [file antibiotics-15-00400-s001.zip › Supplementary material Figures S1 and S2.pdf]

Supplementary Material Figure S1 and S2 Raw Images

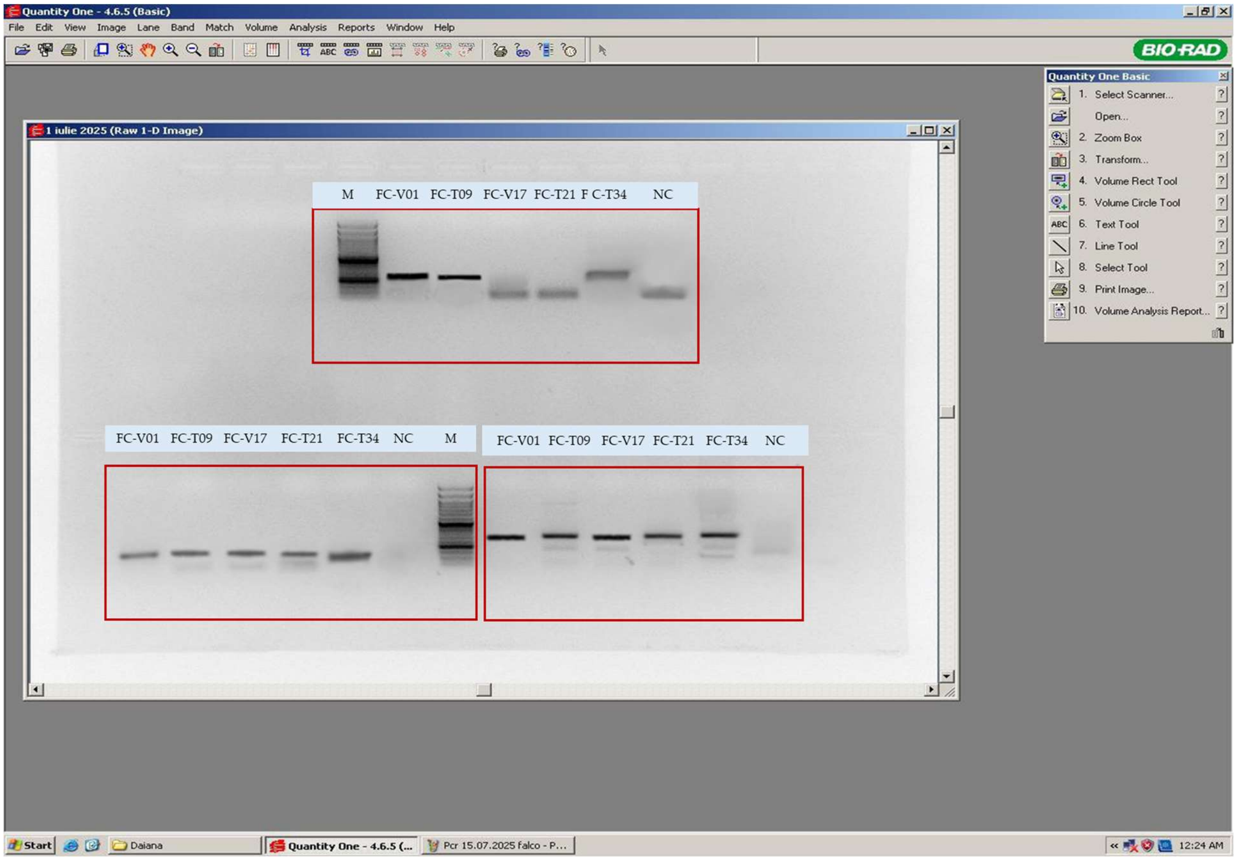

Figure S1. Uncropped Figure 3. Agarose gel electrophoresis of PCR products showing amplification of selected antimicrobial resistance genes in Gram-negative isolates from Saker Falcons (*Falco cherrug*). **Lane M:** 50 bp DNA Ladder RTU (Simply). **(a) Lanes FC-V01, FC-T09, FC-T34:** amplification of the *tetK* gene, showing distinct bands at approximately 360 bp; **(b) Lanes FC-V01, FC-T09, FC-V17, FC-T21, FC-T34:** detection of the *blaZ* gene with an expected amplicon size of 173 bp; **(c) Lanes FC-V01, FC-T09, FC-V17, FC-T21, FC-T34:** amplification of *the ampC* gene with bands corresponding to ~334 bp. **Lane NC**: internal negative control (DNA from pansusceptible isolate), showing absence of amplification. Band sizes were estimated by comparison with the 50 bp molecular weight marker.

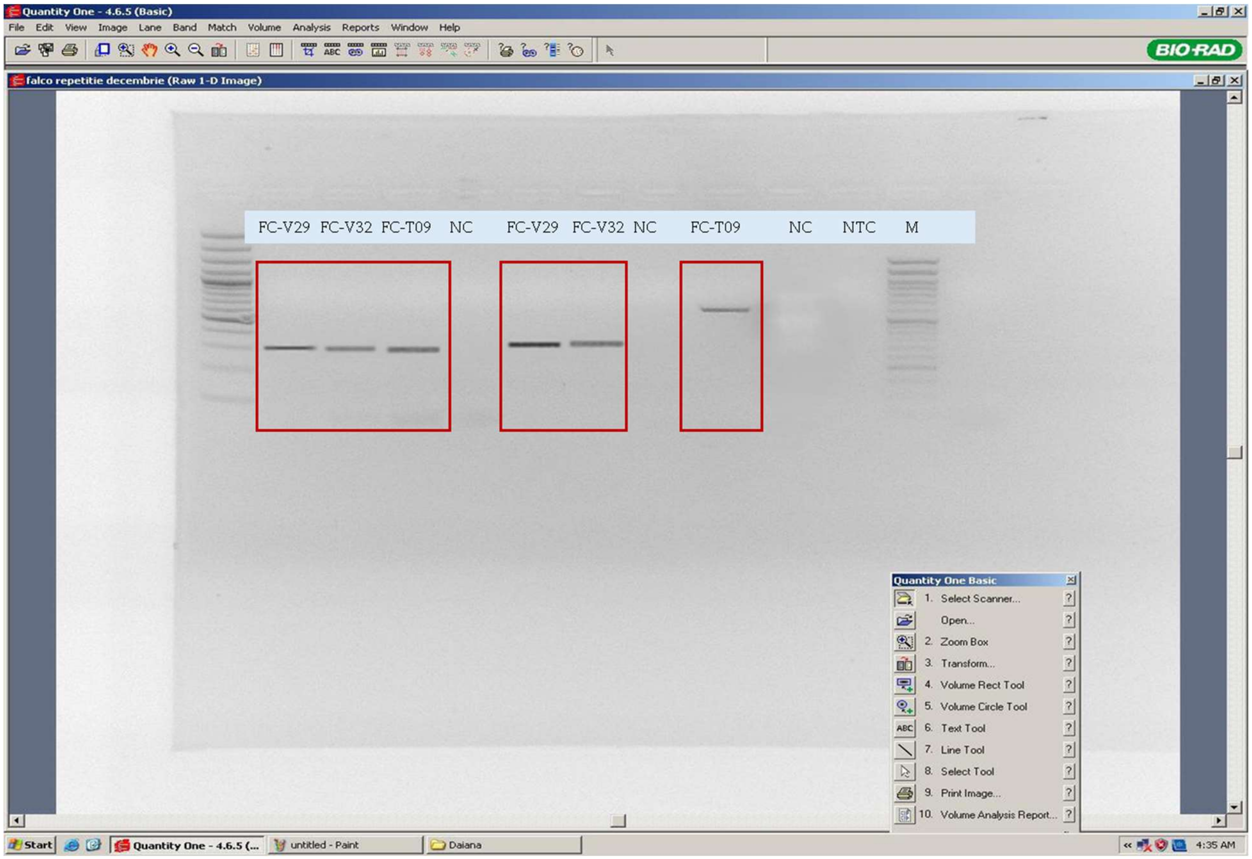

Figure S2. Uncropped Figure 4. Agarose gel electrophoresis showing amplification of fluoroquinolone- and OXA-type β-lactamase-associated genes in isolates from Saker Falcons (*Falco cherrug*). **Lane M:** 50 bp DNA Ladder RTU (Simply). **Lanes FC-V29, FC-V32, FC-T09:** blaOXA-61 amplicon (~280 bp). **Lanes FC-V29, FC-V32:** blaOXA-51 amplicons (~353 bp). **Lane FC-T09:** amplification of the *FQgyrA* fragment with an expected size of 626 bp. **Lane NC:** internal negative control (pansusceptible isolate DNA), showing no amplification. **Lane NTC:** non-template control, confirming absence of reagent contamination. Fragment sizes were determined relative to the 50 bp ladder.
