# Supplementary material for: Antimicrobial Resistance Profiles of Gram-Negative Bacteria Isolated from Saker Falcons (Falco cherrug) in Western Romania
Source: Antibiotics (Basel). 2026 Apr 15;15(4):400. doi: 10.3390/antibiotics15040400 (PMC13114167; doi:10.3390/antibiotics15040400)
Supplement: Supplementary file 1 [file antibiotics-15-00400-s001.zip › Supplementary material Table S1.pdf]

Supplementary Material

Tabel S1. Identification and origin of the 40 bacterial isolates analyzed by the VITEK® 2 automated system.

Crt. no.	Sample ID	Species identified	VITEK ID confidence (%)	Notes
1	*FC-V01*	*Hafnia alvei*	99%	VRBG agar
2	*FC-V02*	*Hafnia alvei*	99%	VRBG agar
3	*FC-V03*	*Hafnia alvei*	99%	VRBG agar
4	*FC-V04*	*Hafnia alvei*	99%	VRBG agar
5	*FC-T05*	*Escherichia coli*	98%	TBX agar
6	*FC-T06*	*Escherichia coli*	97%	TBX agar
7	*FC-T07*	*Escherichia coli*	97%	TBX agar
8	*FC-T08*	*Serratia fonticola*	91%	TBX agar
9	*FC-T09*	*Escherichia hermanii*	98%	TBX agar
10	*FC-T10*	*Escherichia hermanii*	99%	TBX agar
11	*FC-T11*	*Escherichia coli*	99%	TBX agar
12	*FC-T12*	*Escherichia coli*	99%	TBX agar
13	*FC-V13*	*Escherichia coli*	99%	VRBG agar
14	*FC-V14*	*Escherichia coli*	99%	VRBG agar
15	*FC-V15*	*Klebsiella aerogenes*	91%	VRBG agar
16	*FC-V16*	*Escherichia coli*	99%	VRBG agar
17	*FC-V17*	*Escherichia coli*	97%	VRBG agar
18	*FC-V18*	*Escherichia coli*	96%	VRBG agar
19	*FC-V19*	*Escherichia coli*	99%	VRBG agar
20	*FC-V20*	*Escherichia coli*	97%	VRBG agar
21	*FC-T21*	*Escherichia coli*	99%	TBX agar
22	*FC-T22*	*Escherichia coli*	99%	TBX agar
23	*FC-T23*	*Escherichia coli*	97%	TBX agar
24	*FC-T24*	*Escherichia coli*	97%	TBX agar
25	*FC-V25*	*Pantoea agglomerans*	98%	VRBG agar
26	*FC-V26*	*Pantoea spp.*	95%	VRBG agar
27	*FC-V27*	*Pantoea spp.*	96%	VRBG agar
28	*FC-V28*	*Pantoea agglomerans*	98%	VRBG agar
29	*FC-V29*	*Pseudomonas fluorescens*	95%	VRBG agar
30	*FC-V30*	*Pseudomonas fluorescens*	Final ID*	VRBG agar
31	*FC-V31*	*Pseudomonas putida*	Final ID*	VRBG agar
32	*FC-V32*	*Pseudomonas fluorescens*	Final ID*	VRBG agar
33	*FC-T33*	*Escherichia coli*	99%	TBX agar
34	*FC-T34*	*Escherichia coli*	99%	TBX agar
35	*FC-T35*	*Escherichia coli*	99%	TBX agar

36	*FC-T36*	*Escherichia coli*	99%	TBX agar
37	*FC-V37*	*Escherichia coli*	99%	VRBG agar
38	*FC-V38*	*Escherichia coli*	99%	VRBG agar
39	*FC-V39*	*Escherichia coli*	99%	VRBG agar
40	*FC-V40*	*Escherichia coli*	99%	VRBG agar

*Note: In these instances, the software algorithm reached a definitive unique identification (bionumber) without generating a numerical probability score, a documented feature of the VITEK® 2 GN card for certain non-fermenting Gram-negative bacilli when the biochemical reactions matched a single taxon with high specificity.
